# Functional Properties of Extracted Protein from Edible Insect Larvae and Their Interaction with Transglutaminase

**DOI:** 10.3390/foods9050591

**Published:** 2020-05-06

**Authors:** Tae-Kyung Kim, Hae In Yong, Hae Won Jang, Young-Boong Kim, Yun-Sang Choi

**Affiliations:** Research Group of Food Processing, Korea Food Research Institute, Wanju 55365, Korea; privacykin@naver.com (T.-K.K.); awsm_y@kfri.re.kr (H.I.Y.); hwjkfri@kfri.re.kr (H.W.J.); kybaaa@kfri.re.kr (Y.-B.K.)

**Keywords:** edible insects, *Protaetiabrevitarsis*, protein characteristics, functional properties, transglutaminase

## Abstract

Global concern about food supply shortage has increased interest on novel food sources. Among them, edible insects have been studied as a potential major food source. This study aimed to improve the functional properties of protein solutions extracted from *Protaetia* *brevitarsis* (PB) by use of transglutaminase (TG) as a cross-linking agent. After various incubation times (10, 20, 30, 60, and 90 min) with TG, the protein solutions were assessed with regard to their amino acid composition, protein nutritional quality, pH, color (yellowness), molecular weight distribution, thermal stability, foam ability (capacity and stability), and emulsion ability (capacity and stability). Incubation with TG changed the amino acid composition of the proteins and shifted the molecular weight distribution towards higher values, while improving the rest of the aforementioned properties. Since the incubation time for 90 min decreased the protein functionality, the optimum incubation time for cross-linking PB-derived protein with TG is 60 min. The application of TG to edible insect proteins ultimately increases its functionality and allows for the development of novel insect processing technology.

## 1. Introduction

Growing concern about food safety and environmental pollution favors a reorientation of the conventional livestock industry toward environmentally friendly practices and also fuels interest on novel food sources [1,2]. Among the latter are edible insects, as they are considered more environmentally friendly than livestock while possessing a comparable nutritional value [3]. *Protaetia brevitarsis* (PB) has been used for medical purposes in East Asia from ancient times [4]. For these purposes, they are almost always harvested at the larval stage, which is consistent with the results of modern studies investigating the antioxidant activity of PB extracts, as these showed that both the scavenging effect and the phenolic content are higher in extracts from larval-stage than from imago-stage insects [5]. Furthermore, with the exception of a very small amount of Hg, larval extracts contain no harmful components such as bacteria and heavy metals [6].

Besides the antioxidant activity and the safety of raw materials, technical functional properties are crucial as they determine the texture, form, and appearance of food [7,8]. Such properties include foaming capacity, foam stability, emulsifying capacity, and emulsion stability, with the latter two being important to the application of food additives or other ingredients [9]. With respect to these properties, proteins extracted from PB larvae are superior to those extracted from the edible larvae of other insects, namely *Tenebrio molitor* and *Allomyrina dichotoma* [10].

Sometimes, various additives are added to improve the technical functional properties of proteins. Among these, transglutaminase (TG) is considered an excellent binding agent for protein-based food [11]. This enzyme catalyzes the creation of an isopeptide bond by acyl transfer from the γ-carboxamide group of a glutamine residue of a peptide or protein to the γ-amide group of a lysine residue of the same or a different peptide or protein, resulting in a the creation of polymers with improved technical functional properties [12]. Therefore, TG could be a way for enhancing the technical functionality of protein extracted from edible insect larvae. However, despite the aforementioned superiority of the protein extracted from PB larvae in terms of technical functionality, thus far, no study has addressed whether these properties can be further improved by the addition of TG. Therefore, in the current study, we sought to determine the optimal incubation time for processing PB-extracted proteins with TG, using the properties of the produced polymerized protein as the selection criterion.

## 2. Materials and Methods

### 2.1. Materials

PB larvae frozen at the third instar stage (Farm bang, Sunchang, South Korea) and TG (Activa TG-B powder; Ajinomoto Co., Inc., Tokyo, Japan) were obtained from a local market. Reagents used for sodium dodecyl sulfate-polyacrylamide gel electrophoresis (SDS-PAGE) were obtained from Bio-Rad Laboratories, Inc. (Hercules, CA, USA). All other chemical reagents were obtained from Sigma–Aldrich Chemical Co. (St. Louis, MO, USA).

### 2.2. Protein Extraction and Enzyme Reaction

The larvae were freeze-dried at −55 °C for 4 days at 5 Pas in order for the moisture content to decrease to less than 5%. After grounding and screening the dried larvae using 500 μm mesh sieves, 200 g of the larval powder was dissolved in 1000 mL of n-Hexane (99%) and mixed for 1 h at room temperature (20 °C) for defatting then the solvent was removed. After five cycles of defatting, the resulting powder was left under a fume hood for 12 h at ambient temperature (21 ± 1 °C) to remove the residual hexane. The proximate compositions (w/w) of defatted larval powder were measured (moisture: 4.68 ± 0.32%, protein: 64.15 ± 0.52%, fat: 5.84 ± 0.18%, ash: 5.74 ± 0.15%) according to the method of the association of official analytical chemists (AOAC) [13]. To extract the protein, we used a method described in a previous study [10]. Briefly, 200 g of sample was homogenized at 10,000 rpm for 2 min in 400 g of 0.58 M saline solution (0.49 M NaCl, 17.9 mM Na_5_P_3_O_10_, and 1 mM NaN_3_; pH 8.3; 2 °C), and the homogenate was centrifuged at 15,000 g for 30 min at 2 °C. After adjusting the supernatant protein concentration to 1 mg mL^−1^ using the Bradford method [14], TG was added at a concentration of ≈ 0.1 mg mL^−1^. After vortexing, the solution was incubated at 37 °C for various times (10, 20, 30, 60, and 90 min). At the end of each respective incubation period, the solution was heated for 1 min at 80 °C to inactivate the TG. The control sample was heated for 1 min at 80 °C without the addition of TG [15,16]. All subsequent measurements were performed after the solutions were allowed to cool at 20 °C. For each tested incubation time, processing was repeated three times.

### 2.3. Amino Acid Composition

After adding HCl to each sample solution at a final concentration of 6 M, an aliquot of 10 mL was transferred into a sealed ampoule and left to be hydrolyzed under nitrogen at 105 °C for 24 h. The resulting hydrolysate was left to evaporate completely under vacuum at 40 °C, dissolved in 5 mL of 0.02 M HCl, filtered using 0.20 μm membrane filters (GE Healthcare Life Sciences, Pittsburgh, PA, USA), and separated with an ion-exchange resin column (4.6 mm diameter *×* 60 mm length). An L-8800 amino acid analyzer (Hitachi, Tokyo, Japan) was used to determine the amino acid composition of each sample, with results being expressed as mg of amino acid per gram of protein (mg g^−1^) [9]. The acquired values were used to calculate the essential amino acid index (EAAI), which is commonly used as a measure of protein quality [17].

### 2.4. pH Measurement

The pH after the incubation of the protein with TG was measured using a Model 340 pH-meter (Mettler-Toledo GmbH, Schwerzenbach, Switzerland).

### 2.5. Color

Color was evaluated using a CR-410 colorimeter (Konica Minolta, Inc., Tokyo, Japan) with a CR-A50 granular materials attachment, in which 50 mL of sample solution was added. The standard observer (degree, 2), to which all filter functions are adjusted, was based on Illuminant C. Color values were expressed in the CIE 1976 L*a*b*(CIELAB) color space, as dictated by the guidelines of the International Commission on Illumination. Calibration was performed with a white plate (L* 97.83, a* 0.43, and b* 1.98) [18].

### 2.6. Sodium Dodecyl Sulfate-Polyacrylamide Gel Electrophoresis (SDS-PAGE)

SDS-PAGE was used to determine the molecular weight distribution of proteins in the treated and untreated samples [19]. Briefly, 20 μg of each sample was dissolved in 20 μL of loading buffer, mixed well, and heated at 100 °C for 5 min. After cooling, the solutions were loaded in 10% acrylamide Mini-PROTEIN TGX Gels (Bio-Rad Laboratories). Protein bands were stained using Coomassie Brilliant Blue R 250, and their approximate molecular weights were determined using the pre-stained bands of the Precision Plus Protein Dual Color Standards (Bio-Rad Laboratories, Inc.) as a reference.

### 2.7. Differential Scanning Calorimetry (DSC)

DSC was performed to assess the thermal stability of each sample solution [20]. Briefly, an empty aluminum pan and another sealed pan containing 10 μL of sample solution (1 mg mL^−1^) were placed into the internal sensor of a DSC 4000 furnace (PerkinElmer, Waltham, MA, USA), whose temperature range was set to 20–120 °C with a heating rate of 5 °C/min. The onset, peak, and end temperatures, as well as the changes in enthalpy (∆H), were calculated by the Pyris data analysis software (PerkinElmer, Waltham, MA, USA).

### 2.8. Foaming Capacity and Stability

To assess foam ability, 10 mL of each protein solution (1 mg mL^−1^) was homogenized at 12,000 rpm for 2 min using a T-25 ULTRA TURRAX Digital homogenizer (IKA, Königswinter, Germany). The foaming capacity (%) was calculated by dividing the volume of the created foam with the initial volume (i.e., 10 mL) and then multiplying with 100. For assessing foam stability, the “foamed” solution was left to subside, and the foam volume was measured at 2, 5, 10, 20, 30, and 60 min. At each time point, the decrease (%) in foam volume (relative to the initial foam volume) was divided with the initial foam volume and then multiplied with 100 [21].

### 2.9. Emulsifying Capacity and Stability

Emulsions were obtained by the homogenization of a mixture of 10 mL of sample solution and 1 mL of pure olive oil at 18,000 rpm for 2 min. After another 10 min, the volume of the emulsion was measured. The emulsifying capacity was calculated as the percent change between the volume of the emulsion and the initial (pro-mixing) volume, i.e., 11 mL. To assess the stability of the emulsion, 50 μL of the sample was added to 10 mL of 0.3% sodium dodecyl sulfate solution and mixed by inversion. The absorption of the mixed solution was recorded at 0 (i.e., immediately after mixing), 10, 20, 30, 60, 90, and 120 min at 500 nm. For each time point, the percent change between the corresponding absorption and the initial absorption was calculated [22].

### 2.10. Statistical Analysis

All experiments were conducted using triplicate samples. Values are presented as the mean ± standard deviation. A one-way analysis of variance (ANOVA) was performed with IBM SPSS statistics version 20 (IBM Corp., Armonk, NY, USA). Duncan’s multiple range test was used to assess the significance of differences between different TG incubation times, with *p* < 0.05 considered significant. The addition of TG and the chosen reaction times were considered fixed effects. Unconfirmed factors and differences between replicates were considered random effects.

## 3. Results and Discussion

### 3.1. Changes in Amino Acid Composition

Amino acid composition, especially with respect to essential amino acids (EAAs), is the main determinant of the nutritional value of food [17]. In Table 1, the amino acid profile and the EAAI values of each sample of PB-derived protein, non-treated or treated with TG for different times, are presented. Incubation with TG significantly (*p* < 0.05) increased the content of several amino acids. The total amino acid content and the total EAA content were significantly higher (*p* < 0.05) in the treated samples compared to the control sample, while no significant differences among the different incubation times were observed. The higher contents of the treated samples might be due to the increase in the amounts of protein matrix (lysine residue cross linking) created by treatment with TG, as protein solubility and the concentration of protein in solution might be changed by aforementioned linking reactions [12]. The same pattern, i.e., higher values in treated samples but no significant differences among incubation times, was observed for EAAI. However, it is worth mentioning that there were statistically significant increases in the contents of histidine, aspartic acid, and proline as the incubation time increased. Their levels reached a peak after 60 min of reaction time, with further treatment not leading to statistically significant changes.

In conclusion, TG can be used to increase the nutritional value of extracted proteins obtained from edible PB larvae. Moreover, the fact that TG treatment and incubation time increased the content of hydrophobic amino acids, and as a result, the total of these amino acids, implies that the procedure may also improve the technical functional properties of the extracted protein, as hydrophobic amino acids are known to enhance the technical properties of proteins [18]. Further experimentation was performed to confirm this hypothesis.

### 3.2. Changes in pH and Color Values

As seen in Table 2, TG-treated samples had significantly (*p* < 0.05) higher pH values than the control. This might be due to the treatment changing the balance between acidic and basic amino acids. Indeed, incubation with TG had a stronger effect on the basic (>4% increase) than on the acidic (<3% increase) amino acid content, thus making the solution more basic. Importantly, this increase in pH suggests that treatment with TG also increases the technical functional properties of protein, as several studies have shown that basic protein solutions have better technical functional properties than their acidic counterparts [10,21,23].

The color values of a protein solution can be used to estimate the color pigment content of extracted edible insect protein [24]. In our study, CIE L* (lightness) and CIE b* (yellowness) values increased as the reaction progressed, whereas the CIE a* (redness) value decreased (*p* < 0.05). Many factors may lead to changes in the color of protein solutions [18]. TG treatment might have caused the aggregation of color pigment proteins, thus altering their concentrations, or changed the light scattering properties of the protein solution by reducing the space between protein molecules due to cross-linking [25].

### 3.3. Changes in SDS-PAGE Profiles

**Figure 1:**
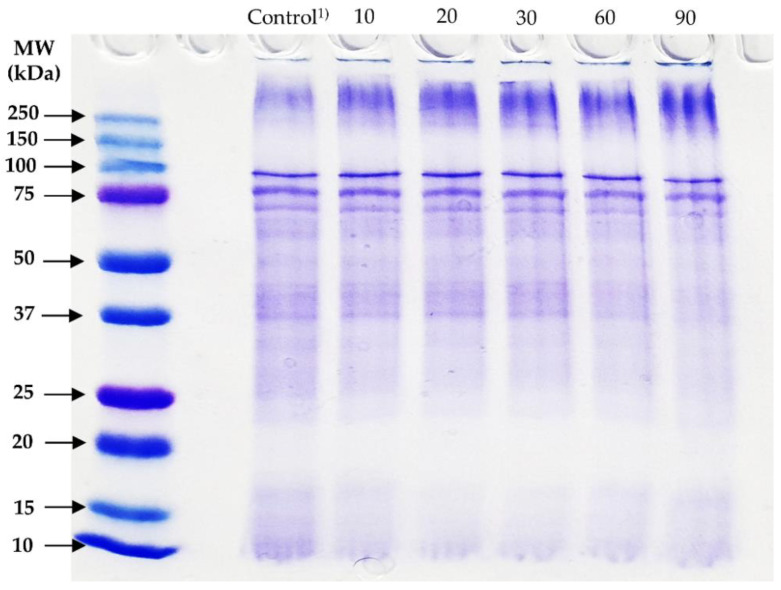
Sodium dodecyl sulfate-polyacrylamide gel electrophoresis (SDS-PAGE) of extracted protein from edible insect interacted with transglutaminase (TG). ^1)^ TG was not added in control and treatment that was added TG was incubated for 10, 20, 30, 60, and 90 min at 37 °C.

SDS-PAGE can be used to assess the extent of cross-linking resulting from TG activity, as it allows the visualization of changes in the molecular weight distribution of the solution’s proteins. As seen in Figure 1, there were clear differences between the TG-treated and control samples. The TG-treated samples had much denser bands in the area corresponding to molecular weights above 250 kDa. Since the used staining dye (Coomassie Brilliant Blue R 250) binds to hydrophobic residues, the high density of protein bands above 250 kDa indicated that hydrophobic residues gathered in high-molecular-weight proteins [26]. In contrast, treated samples had fainter bands under 100 kDa, which can be attributed to these proteins being cross-linked to form larger molecules. Obvious differences also existed among the different incubation times. Thicker bands could be observed at the top of the gel. The protein bands located at the top which suggests that cross-linking for such an extended time started creating molecules too large to enter the gel [12].

### 3.4. Changes in DSC Profiles

Cross-linking due to TG activity increases the thermal stability of proteins, which plays an important role in gel formation [27]. Furthermore, this phenomenon allows the thermal stability of a TG-treated protein solution to be used as indicator of the extent of protein cross-linking [12]. DSC was used to assess the thermal properties of both untreated and TG-treated samples of extracted proteins, and the results are presented in Table 3. All samples showed one endothermic transition, whose onset, peak, and end temperatures tended to increase as the reaction progressed (*p* < 0.05). In contrast, enthalpy (∆H) was lower in the treated than in the non-treated samples (*p* < 0.05). Even though the ∆H fell as time progressed, the differences among the different reaction times were not statistically significant (*p* > 0.05). In contrast, the addition of TG decreased the enthalpy (∆H; *p* < 0.05). This positive relation between TG reaction time and thermal stability is consistent with the findings of a previous study [28]. As no statistically significant changes were observed in any of the three measured temperatures (onset, peak, and end) between 60 and 90 min, the processes of protein aggregation and cross-linking due to TG activity may be considered complete after 60 min of incubation.

### 3.5. Foaming Capacity and Foam Stability

**Figure 2:**
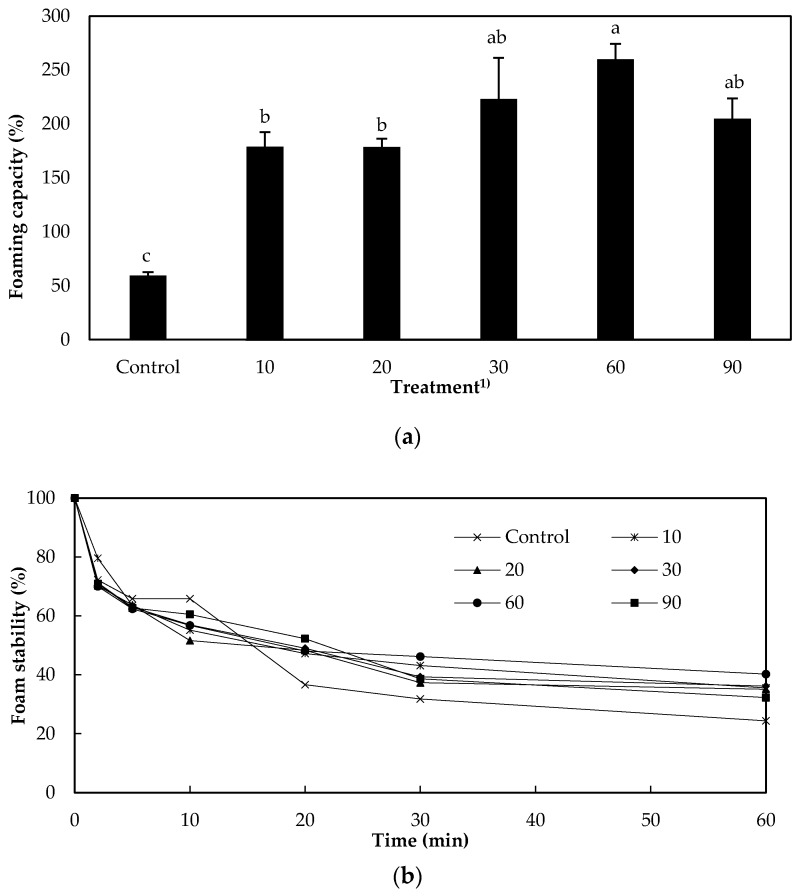
Foaming capacity (**a**) and foam stability (**b**) of extracted protein solution from edible insects interacted with transglutaminase (TG). ^a−c^ Different letters on top of the column meant significantly different (*p* < 0.05). ^1)^ TG was not added in control and treatment that was added TG was incubated for 10, 20, 30, 60, and 90 min at 37 °C.

The foam ability of protein solutions may be broken down into foaming capacity and stability; this factor is important to their use in the food industry [16,17]. As seen in Figure 2a, TG-treated samples had higher foaming capacity than non-treated samples (*p* < 0.05). Among the treated samples, the highest capacity was observed in solutions incubated for 30 min. With respect to foam stability, the long-term stability of the control was lower than that of the treated samples, as suggested by the amount of foam remaining after 30 and 60 min (Figure 2b). Among the treated samples, the best long-term stability was observed in the protein solution that had been incubated with TG for 60 min. In other words, the results for the two foaming-related properties were consistent. The reason underlying the higher foaming capacity and stability of the treated samples may be due to aforementioned reasons. Increased both the hydrophobicity and the pH of the extracted protein solution by TG might be major determinants of its foaming capacity and stability [18,21]. Another factor might be changes in protein solubility due to cross-linking.

### 3.6. Emulsifying Capacity and Emulsion Stability

**Figure 3:**
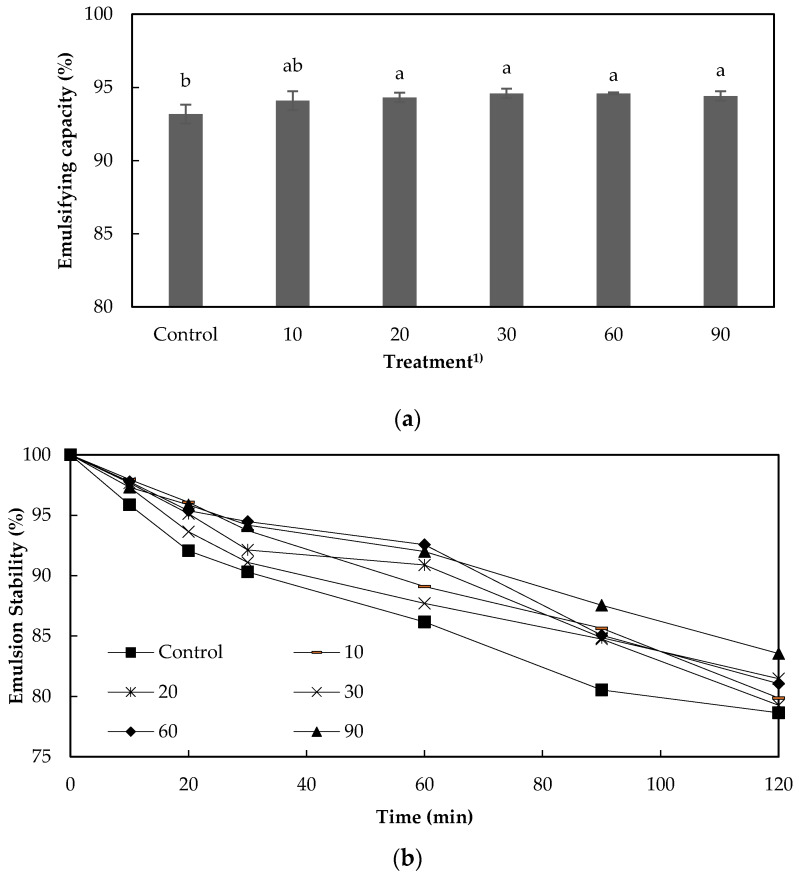
Emulsifying capacity (**a**) and emulsion stability (**b**) of extracted protein solution from edible insect interacted with transglutaminase (TG). ^a–b^ Different letters on top of the column meant significantly different (*p* < 0.05). ^1)^ TG was not added in control and treatment that was added TG was incubated for 10, 20, 30, 60, and 90 min at 37 °C.

The emulsion characteristics of a protein solution, i.e., its emulsifying capacity and the stability of the emulsion, are also very important to use as food resources and these characteristics showed in Figure 3 [29,30]. The emulsifying capacity of the untreated protein solution was over 90%, which is consistent with findings of previous studies on the functionality of proteins extracted from PB [10]. This high emulsifying capacity might result from a high content of hydrophobic amino acids, which could affect positively the interfacial tension [18].

Incubation with TG for 10 min did not affect emulsifying capacity (*p* > 0.05). However, the capacity of solutions produced by longer incubation times was higher than that of the control (*p* < 0.05). With respect to emulsion stability, treatment with TG had a positive effect. With the longest incubation time with TG, the solution with the highest long-term stability and the stability of control was the lowest. This difference in results between the emulsion and foam stability may result from the encapsulation of lipids and lipophilic materials by TG, as this encapsulation might increase the interfacial adsorption ability [12].

## 4. Conclusions

Our study showed that TG treatment had a positive effect on protein extracted from PB, in terms of both nutritional value and functional technical properties. In several properties (protein molecular weight distribution, DSC profile, pH, color, and foam and emulsion ability), differences could also be observed among samples treated with TG for different time durations. These results of the study consequently show the potentiality of protein extracted from PB as food sources or food enhancer for technical properties and their synergistic effect with TG. In most of these cases, an incubation time of 60 min had the peak effect or was sufficient to view the peak effect; therefore, treatment with the enzyme at 37 °C for 60 min seems to be the best choice for processing protein extracted from PB larvae. Increasing protein functionality from PB by using TG could provide a promising insect processing technology and business.

## Figures and Tables

**Table 1:** Amino acid profile and protein quality of extracted protein solution from edible insect interacted with transglutaminase (TG).

Amino Acid Profile (mg/g)	Control ^1)^	Incubation Time (min)					FAO/WHO/UNU ^2)^ (1985)
		10	20	30	60	90	
Essential amino acid (EAA)							
Histidine	12.52 ± 0.30 ^c^	14.73 ± 0.39 ^b^	14.26 ± 0.54 ^b^	14.42 ± 0.03 ^b^	15.63 ± 0.19 ^a^	14.94 ± 0.21 ^ab^	15
Isoleucine	20.57 ± 0.94	19.92 ± 0.33	20.86 ± 1.78	20.69 ± 0.40	20.91 ± 0.92	22.21 ± 1.40	30
Leucine	38.74 ± 1.20 ^b^	44.47 ± 0.03 ^a^	44.45 ± 1.26 ^a^	43.15 ± 0.18 ^a^	45.34 ± 2.37 ^a^	46.40 ± 2.82 ^a^	59
Lysine	7.34 ± 0.17 ^b^	11.88 ± 0.29 ^a^	12.10 ± 0.42 ^a^	11.86 ± 0.38 ^a^	12.12 ± 0.43 ^a^	12.11 ± 0.49 ^a^	45
Methionine + Cysteine	1.12 ± 0.08	2.31 ± 0.49	1.40 ± 0.11	1.85 ± 0.71	2.28 ± 0.26	2.03 ± 0.72	22
Phenylalanine + Tyrosine	61.97 ± 2.74	60.41 ± 5.50	63.08 ± 1.06	58.12 ± 3.48	62.64 ± 1.14	64.09 ± 4.42	38
Threonine	9.24 ± 0.46 ^b^	12.54 ± 0.38 ^a^	12.92 ± 0.38 ^a^	13.42 ± 0.77 ^a^	13.79 ± 0.08 ^a^	13.44 ± 0.69 ^a^	23
Valine	6.74 ± 1.10 ^c^	9.86 ± 0.25 ^b^	9.34 ± 0.96 ^b^	11.01 ± 0.04 ^a^	11.24 ± 0.04 ^a^	11.23 ± 0.27 ^a^	39
Sum of EAA	158.25 ± 5.58 ^b^	176.13 ± 7.60 ^a^	178.42 ± 1.65 ^a^	174.52 ± 5.85 ^a^	183.96 ± 4.84 ^a^	186.46 ± 4.85 ^a^	271
Non-essential amino acid							
Alanine	13.49 ± 0.33	13.99 ± 1.10	13.09 ± 0.06	12.39 ± 0.31	13.57 ± 0.61	13.22 ± 0.03	
Arginine	1.69 ± 0.30 ^b^	4.64 ± 0.78 ^a^	4.53 ± 1.17 ^a^	4.54 ± 0.53 ^a^	4.98 ± 0.78 ^a^	5.65 ± 0.79 ^a^	
Aspartic acid	10.35 ± 0.68 ^c^	15.48 ± 0.63 ^a^	14.53 ± 0.37 ^a^	13.69 ± 0.40 ^b^	15.50 ± 0.90 ^a^	15.98 ± 1.00 ^a^	
Glutamic acid	35.03 ± 0.82 ^b^	46.29 ± 0.45 ^a^	44.30 ± 1.49 ^a^	43.12 ± 1.01 ^a^	45.84 ± 1.23 ^a^	44.98 ± 1.90 ^a^	
Proline	3.38 ± 0.28 ^d^	5.76 ± 0.88 ^c^	7.01 ± 0.40 ^c^	9.25 ± 0.74 ^b^	11.47 ± 0.17 ^a^	11.78 ± 0.06 ^a^	
Glycine	8.90 ± 0.59	7.81 ± 1.08	7.23 ± 0.35	7.59 ± 0.10	7.64 ± 0.12	7.56 ± 0.42	
Serine	18.71 ± 0.60 ^b^	22.60 ± 0.53 ^a^	21.69 ± 0.04 ^a^	22.50 ± 0.69 ^a^	23.10 ± 1.03 ^a^	21.48 ± 1.33 ^a^	
Sum of total AA	249.78 ± 6.82 ^b^	292.69 ± 11.49 ^a^	290.78 ± 0.19 ^a^	287.59 ± 6.80 ^a^	306.07 ± 7.77 ^a^	307.12 ± 10.27 ^a^	
Protein quality	18.39 ± 0.31 ^b^	22.77 ± 0.97 ^a^	21.77 ± 0.37 ^a^	22.36 ± 1.45 ^a^	23.70 ± 0.12 ^a^	23.44 ± 0.76 ^a^	

All values are mean ± standard deviation of three replicates (*n* = 3). ^a−d^ Means within a row with different letters are significantly different (*p* < 0.05). ^1)^ TG was not added in control and treatment that was added TG was incubated for 10, 20, 30, 60, and 90 min at 37 °C. ^2)^ Food and Agriculture Organization/World Health Organization/the United Nations University.

**Table 2:** pH and instrument color of extracted protein solution from edible insect interacted with transglutaminase (TG).

Traits	Control ^1)^	Incubation Time (min)				
		10	20	30	60	90
pH	7.16 ± 0.03 ^b^	7.61 ± 0.06 ^a^	7.62 ± 0.07 ^a^	7.63 ± 0.08 ^a^	7.62 ± 0.10 ^a^	7.63 ± 0.08 ^a^
CIE L* ^2)^	15.39 ± 0.03 ^d^	16.20 ± 0.01 ^c^	16.28 ± 0.10 ^bc^	16.30 ± 0.09 ^ab^	16.29 ± 0.09 ^ab^	16.37 ± 0.02 ^a^
CIE a*	2.08 ± 0.10 ^a^	1.75 ± 0.13 ^b^	1.79 ± 0.10 ^b^	1.86 ± 0.15 ^b^	1.79 ± 0.11 ^b^	1.80 ± 0.11 ^b^
CIE b*	0.48 ± 0.10 ^c^	0.49 ± 0.07 ^bc^	0.49 ± 0.05 ^bc^	0.45 ± 0.05 ^bc^	0.56 ± 0.05 ^ab^	0.62 ± 0.04 ^a^

All values are mean ± standard deviation of three replicates (*n* = 3). ^a−d^ Means within a row with different letters are significantly different (*p* < 0.05). ^1)^ TG was not added in control and treatment that was added TG was incubated for 10, 20, 30, 60, and 90 min at 37 °C. ^2)^ International Commission on Illumination (CIE) L*, CIE a*, and CIE b* meant lightness, redness, and yellowness.

**Table 3:** Differential scanning calorimeter of extracted protein solution from edible insect interacted with transglutaminase (TG).

Traits	Control ^1)^	Incubation Time (min)				
		10	20	30	60	90
Onset temperature ^2)^	36.03 ± 0.03 ^d^	40.03 ± 0.01 ^d^	49.50 ± 1.89 ^c^	55.06 ± 3.91 ^b^	58.44 ± 0.71 ^ab^	60.72 ± 1.29 ^a^
Peak temperature	47.86 ± 1.20 ^d^	52.37 ± 3.28 ^c^	57.66 ± 1.41 ^b^	60.28 ± 1.77 ^ab^	62.35 ± 0.71 ^a^	63.82 ± 0.01 ^a^
End temperature	61.33 ± 0.61 ^d^	63.88 ± 0.22 ^c^	66.85 ± 0.12 ^b^	67.53 ± 0.03 ^b^	68.89 ± 0.81 ^a^	68.56 ± 0.07 ^a^
∆ H	2.52 ± 0.72 ^a^	0.89 ± 0.01 ^b^	0.73 ± 0.10 ^b^	0.60 ± 0.02 ^b^	0.33 ± 0.08 ^b^	0.22 ± 0.08 ^b^

All values are mean ± standard deviation of three replicates (*n* = 3). ^a−d^ Means within a row with different letters are significantly different (*p* < 0.05). ^1)^ TG was not added in control and treatment that was added TG was incubated for 10, 20, 30, 60, and 90 min at 37 °C. ^2)^ The unit of temperature is °C and enthalpies (∆ H) unit is J g^1^.
